# Family management risk and protective factors for adolescent substance use in South Africa

**DOI:** 10.1186/s13011-018-0163-4

**Published:** 2018-06-19

**Authors:** Beatrice Wamuyu Muchiri, Monika M. L. dos Santos

**Affiliations:** University of South Africa, Pretoria, South Africa

**Keywords:** Risk factors, Protective factors, Substance use, Adolescents, Family management, Discipline and behavioural control, Parental monitoring, Parental discipline, Parental rewards, South Africa

## Abstract

**Background:**

An increasingly recognised prevention approach for substance use entails a reduction in risk factors, and the enhancement of promotive, or protective factors in individuals and the environment surrounding them during their growth and development.

**Methods:**

This exploratory study evaluated the effect of potential risk and protective factors associated with family management relating to adolescent substance use in South Africa. Exploratory analysis and cumulative odds ordinal logistic regression modelling was performed on the data, while controlling for the influence of demographic and socio-economic characteristics on adolescent substance use.

**Results:**

The most frequently used substances were cannabis, followed by other illicit substances and alcohol in decreasing order of use intensity. The specific protective, or risk effect of family management factors, varied according to substance. Risk factors associated with demographic and socio-economic factors included being male, of a younger age, lower education grades, of a coloured ethnicity, adolescents from divorced parents, and unemployed or fully employed mothers. Several family management factors, categorised as parental monitoring, discipline, behavioural control and rewards, demonstrated either risk or protective effects on adolescent substance use.

**Conclusions:**

This exploratory study demonstrated that various risk and protective factors associated with family management may affect adolescent substance use. Interaction amongst risk or protective factors, as well as the type of substance, should be considered when further considering interventions based on these risk or protective factors.

## Background

Substance use among adolescents has been reported to significantly affect the health and various facets of individual well-being [1]. With close to half of the South African population consisting of youth 20 years old or younger [2], it is important to pay attention to the use of Alcohol and other Drugs (AODs) by this group due to the potential implications for the country’s socio-economic development [3].

Negative health consequences are increasingly being addressed by prevention science, which involves reducing risk and enhancing promotive or protective factors in individuals and the environment surrounding them during their growth and development [4]. Risk factors predict enhanced likelihood of problems, while protective factors mediate or moderate exposure to the risk [5]. Protective factors buffer adolescents from exposure to risks leading to a reduced likelihood of acquiring such problematic behaviours [6]. Additionally, promotive factors play a further role in the decreased likelihood of health problems [7]. Protective factors are distinguished from promotive factors because the later moderates the negative effects of risks for predicting negative outcomes, and therefore only compensates for risk exposure [8]. An understanding of these risk and protective factors is important in the development of effective interventions. Risk and protective factors affecting substance use can be categorized as contextual, variable and individual risk, and protective factors - which have been extensively reviewed [1]. Fixed markers include gender, biological indicators, income, family substance history, parent psychopathology, parental marital status and income/ social economic status. Contextual variables include factors such as law, availability, social norms and community order. Examples of individual variables incorporate family relations, family management, education factors, a positive attitude or expectancies, social competence, peer relations, religious involvement, conformity or moral order, living situation, stressful events, individual psychopathology and adolescent substance use [1]. Among individual and interpersonal risk and protective factors, family environment influences the likelihood of substance abuse problems significantly. Family environment is viewed in terms of family relations and family management [1]. Modification of risk and protective factors may ameliorate harms from substance abuse prior to birth, and continue through to young adulthood. These developmental periods are predominantly spent in the family context [1].

Family relations and their influence on substance use can be viewed either in terms of connectedness or conflict [1]. Increase in either parent to parent conflict, or parent to offspring conflict, has been shown to increase the risk of developing a substance use disorder [9]. The level of family bonding and support by parents to their offspring are a predictor of alcoholism and drug use amongst the youth [1]. Favourable family bonds or relationships may also reduce the likelihood of substance use problems, even amongst those with personality problems [10]. The social development model postulates that children learn behavioural patterns from their social environment - including family, school, peers and community institutions either in a pro or an antisocial pathway. The dynamic nature of society and new trends in substance use necessitate the identification of risk factors as an on-going process. Treatment programmes and models too should be revised according to the patterns of risk elements in different cultures and social groups in society [11]. Mitigation measures are not universal and risk factors are influenced by cultural groupings which have called for culturally relevant programmes [12]. An increasing number of studies have therefore identified factors influencing substance use in industrialised nations, however, there are few studies in South Africa and other developing countries that explore these facets [12].

From a review of published literature, it is evident that there is a general lack of studies focusing on family predictors of substance use based on family management and relations. Brook et al. [12] assessed the effect of two types of parental factors in South Africa: parental drug use and adolescent’s identification with the parent. However, no investigators have focused on how family factors, aside from the parent–child relationship, predict adolescent substance use in South Africa and other developing countries [12]. These factors, and their interactions, would provide more insight into possible family environment based intervention strategies. Such interactions include risk, through to protective interaction (for example, risk factor of family substance drug use being ameliorated by a good family environment, leading to less drug use), to protective factor interactions (for example, the protective factor of low family substance use being enhanced by good family environment, leading to less drug use).

The study of risk and protective family management risk and protective factors for adolescent substance use is projected to support evidence based treatment and intervention programmes by policy makers. Treatment and intervention programmes and studies should account for the patterns of risk elements in different cultures and social groups in society [11, 13]. Such programmes can be founded based on the social development model, which is a theory of causation and prevention, and an important prerequisite to an intervention strategy seeking to mitigate risk factors, while at the same time enhancing protective factors [5]. Theory-driven intervention elements based on this model include (i) creation of opportunities for pro-social activities for the adolescents; (ii) offering of empowerment towards successful performance of these activities; and (iii) offering positive reinforcement for successful contribution. Protective factors buffer adolescents from exposure to risks and reduce the likelihood of acquiring such behaviours [5, 13].

This study offers a pilot exploration of important family management risk and protective factors that affect alcohol and other drugs use problems amongst adolescents in South Africa.

## Methods

### Participants

The principal investigator personally interviewed adolescent participants with a history of substance use. Participants were sourced from rehabilitation centres in Pretoria, namely Staanvaas and Castle Carey Clinic, between September 2014 and June 2015, and were contacted upon ethical approval of the study. Ethical approval was provided by the Ethics Committee of the Department of Psychology at the University of South Africa (UNISA) with special reference to the requirements of the Code of Conduct for Psychologists of the Health Professions Council of South Africa (HPCSA) and the UNISA Policy on Research Ethics. The 54 respondents consisted of 48 males and six females between the ages of 14 and 20 years from different socio-economic backgrounds. Respondents were selected by stratified random sampling, with rehabilitation centres serving as the stratum.

### Procedure

Data was collected by using a structured pre-tested paper based questionnaire after acquisition of informed consent from the institutional directors, parents or guardians of the adolescents below age 17, as well as the adolescents. Both the respondent and interviewer swapped booklets, and both marked the responses directly on the questionnaire. The interviewer then proceeded to cross-check the responses after the interview. Table 1 displays the variables and components of the scales used.

**Table 1:** Family relations and management variables and their measures

Variable	Measures	Reference
Background Variables	Gender, age, level of education, cultural background, parental marital status, parental education, parental socio-economic status	[1]
Family Management		[1]
Parental monitoring	Monitoring	[15]
	Delinquency	[16]
Discipline and Behavioural Control	Sharing, control through guilt, strictness, expression of affection, emotional support, parental direction, sharing, moderate autonomy, lax discipline, positive evaluation, negative evaluation, irritability, extreme autonomy, laissez-faire family style	[17, 18]
Parental rewards	Good behaviour, achievement,	[31]
Substance use		
Adolescent and parental substance use	Intensity and frequency of alcohol use, intensity and frequency of alcohol use, frequency of other substance use, age at initiation of use	[14]

### Measures

#### Background variables

Background variables (or socio-economic variables) such as ethnicity, gender, parental education, parental marital status and income / socioeconomic status have been shown to influence substance use and abuse [1]. These background variables were factored in during data analysis, and variables influencing the results significantly were controlled for their effect on study variables.

#### Youth and parental substance use

The frequency and intensity of which alcohol, cannabis, and other illicit drugs are used were measured. The frequency of illicit drug use was measured using an open ended questionnaire that explored over the preceding two years the non-medical and purpose of use. Illicit drug used amongst participants include amphetamines, barbiturates, cocaine, heroin, LSD or other psychedelics and tranquilizers. Response categories included: 7 = everyday or almost every day; 6 = 3 to 5 days a week; 5 = 1 or 2 days a week; 4 = 2 or 3 days a month; 3 = once a month or less; 2 = 1 or 2 days in the past 12 months; 1 = never [14]. Response categories for intensity included the number of substance units used where categories included: 1 = none; 2 = 1 or 2; 3 = 3 or 4; 4 = 5 or 6, 5 = 7 or 8, 6 = 9 or 10; 7 = 10 or more.

#### Family management

Family management is a broad concept which encompasses (i) parental monitoring, (ii) discipline, (iii) behavioural control, and also (iv) the reward system set in place by parents to reinforce good behaviours [1].

Parental monitoring was assessed using the parental monitoring measurement tool developed by Arria et al. [15] consisting of nine questions on a five point scale, namely: “level 1 = never”, “level 2 = rarely”, “level 3 = sometimes”, “level 4 = often” and “level 5 = always”. Adolescents were asked to recall their high school experiences and rate on a four point scale responses to questions such as: i) when one gets home from school, how often was an adult there within an hour of you getting home, ii) when one went to parties, how often was a supervising adult present at the party and iii) when one wanted to go to a party, how often did parents confirm that an adult would supervise the party. This tool was modified to include predictors of delinquency in adolescents as proposed by Steinberg et al. [16]. The scale contains items that include questions on the child’s perception of parental rule-setting, supervision, consequences and monitoring which were scored on five-point scale per item.

Parental discipline and behavioural control was measured using the Children’s Report of Parental Behaviour Inventory [17] that assesses the consistency of discipline and rule enforcement (30 items each for both the mother and father). Correlation coefficients were analysed between maternal and paternal support. The use of power-assertive techniques by parents to control their children was also measured as the sum of paternal and maternal scores on the five-item maternal and paternal discipline scales [18]. Discipline and behavioural control was measured either as “level 1 = not like”, “level 2 = somewhat like” or “level 3 = a lot like”. The higher the score, the greater the degree of disciplinary measures used. Parental rewards was measured by asking how often parents rewarded good behaviour and achievement and responses were: “level 1 = never”, “level 2 = often” and “level 3 = always”.

### Data analyses

Frequency and intensity of adolescent alcohol use were used to compute legal substance use. Combined scores representing “other illicit substance use” were calculated from the frequency of the use of amphetamines, barbiturates, cocaine, heroin, LSD or other psychedelics, tranquilizers and other substances. Parental substance use was calculated either as legal substance (alcohol) use, or illicit substance use with combined scores from the rest of the substances. Reliability of the different dimensions or constructs in the questionnaire is indicated by their Cronbach’s Alpha values [19]. Items of the scale were either retained or removed based on their Cronbach Alpha values [19].

Exploratory data analysis was performed by cross tabulation of predictor and response variables and exploration of their interrelations. Cumulative odds ordinal logistic multivariate regressions with proportional odds were run to determine the effects of family management and relation variables controlling for demographic and socio-economic characteristics on adolescent substance use. Modelling was first performed for each independent variable against adolescent alcohol, cannabis and other illicit substance use. Variables that were significantly different at a screening *p*-value ≤0.1 were entered into multiple ordinal logistic regression models controlling for significant demographic and socio-economic characteristics [20]. The model was further considered to be of statistical significance insofar an association with the dependent variable over and above the intercept-only model whenever *p*-values were ≤ 0.05. Adjusted odds ratios with *p*-values and 95% confidence intervals were obtained to compare the influence of the family characteristics.

In the second part of the study, all family management variables and controlled variables were incorporated into a single logistic regression model for each of the descriptors of the family management variables in an exploratory manner. A backward elimination was applied to remove those variables with less explanatory power towards the substance use, according to their *p*-values. The final model was one in which all remaining family factors were significant [21, 22].

The proportional odds assumptions were assessed using a full likelihood ratio test comparing the fitted model to a model with varying location parameters where *p*-values greater than 0.05 are considered acceptable. Deviance and Pearson goodness-of-fit tests were performed with an indication that the model was a good fit to the observed data whenever *p*- value was greater than 0.05.

## Results

Table 1 reports all the studied constructs. After variables selection and dropping of most of the variables from the final models, this section as well as Tables 2 and 3 outlines statistically significant results of the specific items of the constructs that are measures of the broad study constructs.

**Table 2:** Results from ordinal logistic regression predicting substance use in adolescents given demographic and socio-economic characteristics

Variable		Substance	Odds (95% CI)	Model Fit	*p*-value
Gender		Cannabis	5.035 (1.012–25.05)	χ2(1) = 3.9	0.048
Age		Cannabis	0.738 (0.536–1.016)	χ2(1) = 3.968	0.046
Ethnicity					
	Coloured versus white	Alcohol	15.637 (2.880–84.9)	χ2(1) = 10.149	0.001
	Coloured versus black	Alcohol	13.578 (2.763–66.735)	χ2(1) = 10.310	0.001
Parental Employment	Maternal				
	Unemployed versus self employed	Cannabis	15.449 (1.398–170.8)	χ2(1) = 4.987	0.026
	Full time employed versus self employed	Cannabis	12.764 (1.331–122.4)	χ2(1) = 4.876	0.027
	Part time employed versus self employed	Illicit	28.888 (1.251–66.18)	χ2(1) = 4.409	0.036

**Table 3:** Results from ordinal logistic regression predicting substance use in adolescents given family management variable parental monitoring

Family management variable	Measure	Substance	Cronbach Aplha	Odds (95% CI)	Model Fit	p-value
Parental monitoring	Parental knowledge	Alcohol	0.84	0.556 (0.312 to 0.991)	χ2(1) = 3.964	0.046
	Adolescent recall	Illicit substance	0.84	0.428 (0.238 to 0.975)	χ2(4) = 11.323	0.023
Discipline and behavioural control	Sharing	Alcohol	0.73	6.447 (1.642 to 25.313)	χ2(1) = 7.131	0.008
	Control through guilt	Alcohol	0.6	12.782 (1.418–115.217)	χ2(1) = 5.159	0.023
	Strictness	Alcohol	0.82	3.646 (1.204–11.039)	χ2(1) = 5.239	0.022
	Affection	Alcohol	0.75	3.349 (1.092–10.275)	χ2(1) = 4.467	0.035
	Emotional support	Cannabis	0.85	3.7 (0.966–14.16)	χ2(1) = 3.648	0.05
	Positive evaluation	Cannabis	0.87	3.723 (1.027–13.492)	χ2(1) = 4.005	0.045
	Negative evaluation	Illicit substances	0.64	0.184 (0.028–1.192)	χ2(4) = 10.176	0.038
Rewards	Parental rewards	Alcohol	0.72	4.164 (1.133–15.302)	χ2(1) = 4.616	0.032
Parental use	Parental legal substance	Illicit substance	0.78	0.108 (0.012–1.000)	χ2(1) = 3.841	0.05
	Parental illicit substance	Alcohol	0.88	0.073 (0.010–0.525)	*χ*^2^(1) = 6.751	0.009

### Demographic and socio-economic characteristics

The odds of cannabis use by males were statistically higher and 5 times that of females. An increase in age of the adolescents was associated with 1.4 times decrease in odds of higher cannabis use (Table 2).

Alcohol use significantly differed according to adolescent ethnicity, whereby the odds of higher frequency of alcohol use for colored respondents was 16 times and 14 times higher than that of white and black respondents respectively (Table 2). The odds of higher frequency of cannabis use for adolescents from unemployed and full time employed mothers were 16 and 13 times higher than those from self-employed mothers. The odds of illicit substance use for adolescents from part time employed mothers were 29 times higher than those from self-employed mothers (Table 2).

### Family management outcomes

The results from ordinal logistic regression assessing the effect of family management variables on adolescent substance use are presented in Tables 3. This section presents those results that were statistically significant.

### Parental monitoring

Table 3 shows results from ordinal logistic regression predicting substance use in adolescents with changes in parental monitoring. The odds of using alcohol more frequently with parental monitoring as measured by parental knowledge of adolescent activities were 3.9 at the lowest category than those of level 3.

Parental monitoring as measured by parental knowledge of adolescent activities controlling for ethnicity significantly predicted higher adolescent alcohol use (Table 3). The final model significantly explained the dependent variable over and above the intercept-only model. The odds of using alcohol more frequently indicated a 1.8 times decrease in odds of using alcohol more frequently with each increase in the level of parental knowledge of adolescent activities.

Effect of after school parental monitoring on use of other illicit substance was tested controlling for maternal employment status. The final model statistically significantly predicted the dependent variable over and above the intercept-only model (Table 3). The odds of using alcohol more frequently decreased 2.3 times with every increase in parental knowledge of adolescent activities.

### Discipline and Behavioural control

Table 3 displays results from ordinal logistic regression predicting substance use in adolescents as influenced by discipline and behavioural control.

### Discipline and behavioural control against alcohol use

Sharing, control through guilt, strictness and affection statistically significantly predicted adolescent alcohol use even when ethnicity was controlled for.

The odds of consuming alcohol more frequently when sharing was at lowest category sharing were 6.5 times than when sharing was at the second higher category. The odds of being in a higher category of alcohol use when behavioural control through guilt was at category were 12.8 times when compared to level 2 (Table 3).

The lowest category of parental strictness increased the odds ratio of more frequent consumption of alcohol by 3.7 times more than when strictness was in category 2. The odds ratio of being in a higher frequency of alcohol consumption when affection was at lowest category was 3.4 more than when affection was at category 2 (Table 3).

### Discipline and behavioural control against cannabis use

Adolescent cannabis use as influenced by emotional support and positive evaluation was assessed controlling for gender, age, marital status of parent and maternal employment status. The odds of higher frequency of using cannabis when emotional support was at lowest level were 3.7 times more than those when emotional support was at level 2. The odds of using cannabis more frequently when positive evaluation was at lowest were 3.7 more than when positive evaluation was at level 2 (Table 3).

### Discipline and behavioural control against other illicit substance use

The odds of adolescents using illicit substances more frequently when negative evaluation was at the lowest level were 5.3 times than when negative evaluation was at level 2.

The effect of negative evaluation by parents on adolescent illicit substance use was assessed, controlling for maternal employment status. The final model significantly explained maternal employment status over and above the intercept-only model. There was a 5.4 decrease in the frequency of illicit substance use with each unit increase in negative evaluation.

The effect of discipline and behavioural control on adolescent cannabis use was tested controlling for gender, age, marital status of parent and maternal employment status. The final model indicated that discipline and behavioural control when maternal employment and marital status is controlled for did not statistically significantly predict higher adolescent illicit substance use.

### Parental rewards

Table 3 depicts results from ordinal logistic regression predicting the influence of parental rewards on substance use in adolescents. The odds of using alcohol more frequently when parental rewards were rated at lowest category were 4.2 times more than when parental rewards was at category 3. The effect of parental rewards on adolescent alcohol use was assessed controlling for ethnicity. However, parental rewards did not statistically significantly predict higher adolescent alcohol use when ethnicity was controlled for.

### Parental substance use

Results from the ordinal logistic regression assessing the effect of parental substance use on adolescent substance use are presented in Table 3. When parental legal substance use was considered, there was a 13.7 and 9.26 decrease in adolescent illicit substance at the lowest parental legal substance use categories 1 and 2 respectively when compared with parental legal substance use category 6.

Considering the influence of parental legal substance use on adolescent illicit substance use controlling for ethnicity, the model statistically significantly predicted higher adolescent alcohol substance use. The odds of being in a higher category of alcohol use increased 1.5 times with each increase in the category of parental illicit substance use.

## Discussion

The age of respondents in this study ranged between 14 and 20. This stage is characterized by a rapid change to a new social phase where individuals have greater freedom and less social control when compared to the experience during childhood [1].

Cannabis was the most highly used illicit substance as reported by 63% of the adolescents. This may be a reflection of a higher societal tendency towards an acceptance of cannabis use in comparison to other illicit substances of abuse, though cannabis use might be associated with more deviance among adolescents and adults users than those who do not initiate use [23, 24].

Study outcomes suggest that the increased alcohol use by parents was a risk factor for illicit substance use by adolescents. Risk factors associated with demographic and socio-economic factors for substance use among the adolescents included being male, younger age, being in lower education grades, coloured ethnicity, adolescents from divorced parents and unemployed or fully employed mothers. Such factors are fixed implying that they cannot demonstrate change but mitigation efforts can be focused on adolescent demographic groups in categories at higher risk [1].

Results further indicate a relationship between the working status of mothers and the risk of cannabis as well as other illicit substance use. Controlling for maternal employment status also resulted in changes in the significance of the relationship of other variables with substance use. This should be interpreted with caution due to the fact that caregiving in mainly maternal in South Africa, which may moderate the maternal care availability versus adolescent alcohol use. Primary caretakers of children in South Africa are predominantly female, and at least 92% of primary caretakers of children in poor households are females. Further evidence can be derived from child support grant system where the primary caregiver of the minor child receives the grant regardless of their gender. In this respect, studies from the initial years of the grant recipients indicates that only 0.2% of the caretakers were men, though this has slightly increased to 3–8%. However, behavioral problems in childhood and later in life, especially in adolescence, have been in many studies associated with mothers who are distant emotionally or physically [25, 26]. Furthermore, severe maternal deprivation has been regarded as a key contributor to juvenile delinquency [27]. Alternative caregiving by nannies often leads to exposure to different caretakers which has also been associated, especially during the first few years, with antisocial behaviour [28]. However, the quality of alternative care, especially daycare rather than the effect of maternal care, may be more important. Hausfather, Toharia, LaRoche, and Engelsmann [29] for instance report beneficial effects of longer-term exposure to high-quality child care centers, but detrimental effects of longer-term exposure to poor-quality child care centers, with respect to noncompliant behaviour in children.

The history of family management may predict current substance use [30]. The significance of various factors in this study varied with the type of substance. These factors were classified as parental monitoring, discipline, behavioural control and rewards.

Demographic and socio-economic factors associated with increased substance use among the adolescents included being male, younger age, being in lower education grades, coloured ethnicity, adolescents from divorced parents and unemployed or fully employed mothers.

### Parental monitoring

Low parental monitoring was associated with increased likelihood of engagement in alcohol use in adolescents. In similar results, a study of eight to ten year old children over a three-year period reported a 1.6-fold reduction in substance use initiation with increased levels of parental monitoring and supervision [15].

Even when age was controlled for, the odds of using alcohol more frequently decrease with increasing parental knowledge of adolescent activities. Increased parental monitoring of adolescent activities was also associated with decreased illicit substance use. Childhood and adolescent risk of later alcohol abuse and dependence may be reduced and protection enhanced by early establishment and maintenance of close parental or other adult monitoring and supervision activities [15, 31, 32]. More parental monitoring and supervision also leads to a delay in substance use initiation, as well as less frequency and intensity of substance use [14, 15, 33, 34]. Lastly, enhanced parental monitoring and supervision is correlated with less high school alcohol consumption, independent of gender, ethnicity and religiosity [15].

### Discipline and Behavioural control

Parental sharing and control through guilt and affection were significantly associated with adolescent alcohol use even when ethnicity was controlled for. Adolescents whose parents scored low in sharing were more likely to use alcohol than those with more sharing parents. Less employment of behavioural control through guilt by parents was associated with more likely to use alcohol. A similar trend was observed for parental strictness, where less parental strictness was associated with increased consumption of alcohol. Adolescents who received less affection from parents were more likely to use alcohol than those receiving more affection. This implies that among factors related to discipline and behavioural control, risk factors influencing substance use included lower levels of sharing, control through guilt, parental strictness, affection, emotional support, positive evaluation and negative evaluation. Decision making by parents, setting of rules and limits, as well as monitoring and defining behavioural control - which is a socialisation dimension associated with reduced adolescent substance use, deviance and engagement in early sexual intercourse - were also highlighted as key risk aspects [14]. Parental permissiveness to substance use in childhood or early adolescence also increases the risk of early age initiation of substance use [33]. Parent-child interactions devoid of closeness influence substance initiation and they are a predictor of substance use. Emotional and inter-personal sharing, on the other hand, offers a protective effect as it supports the growth of adolescents in families characterized by feelings of parental trust, warmth, and involvement [35].

Higher levels of behavioural control through guilt and strictness were associated with less adolescent alcohol use. Parental strictness is most firmly associated with lessened youth antisocial behaviour when compared to other major protective aspects against youth antisocial behaviour including positive peer relations and behavioural control [14]. Clear censure of underage drinking has been reported among other effective parenting practices with an effect on adolescent drinking reduction [15].

Lower affection received from parents was associated with increased alcohol use and affection showed an interactive effect with sharing and behavioural control through guilt. Nurturance/warmth and demands for responsible behaviour have been found to be important determinants of effect of parenting. High nurturance and more demands by parents lead to more authority, which is a predictor of better developmental outcomes in children [33]. Indirect control, which involves parent-child closeness, may have a significantly higher effect on the prevalence of delinquent behaviour than direct control involving parental involvement and monitoring [6].

An increase in parental emotional support and positive evaluation was associated with decreased intensity of cannabis use in adolescents. Conversely, adolescents were more likely to use cannabis when they received less positive support by parents. There was a decrease in the frequency of illicit substance use with increased negative evaluation. Among reported parental socializing practices associated with less substance use and other adolescent deviant behaviours include: emotional and instrumental support, as well as moderate levels of control [34]. Parental socialisation aspects nurturing positive behavioural development in adolescents include positive evaluation through enhanced autonomy [14].

Increased adolescents negative evaluation by parents was associated with a decrease in the frequency of illicit substance even when maternal employment status was controlled for. In this study, negative evaluation at higher levels, therefore, appeared to have a protective effect on adolescent substance. This effect may be explained by a possible similar effect to that of discipline and behavioural control measures [36].

In conclusion, discipline and behavioural control, nurturance of behaviour, creating boundaries and the setting of clear rules, are some of the universal prevention strategies within the family that may be employed to reduce incidences and onset of delinquency, including substance abuse through family based interventions [6].

### Parental rewards

Lower levels of parental rewards were associated with higher risk of alcohol use. However, when the effect of ethnicity was controlled for, parental rewards were not significantly associated with higher adolescent alcohol use. The risk of later childhood and adolescence alcohol abuse and dependence may be reduced and protection enhanced by providing appropriate parental rewards for good behaviour in children [31]. Conversely, family management typified by limited and inconsistent rewards for positive behaviour is characterized by increased risk of substance use, violence, and delinquency [32].

### Parental substance use

Lower parental legal substance (alcohol) use had a protective effect against higher illicit substance use among the adolescents. Prior evidence indicates that children develop positive attitudes about alcohol use when their parents, or other family members, drink more and hold positive alcohol-related expectancies [15, 31, 36]. Conversely, adolescents whose parents have negative attitudes toward alcohol and disapprove of underage drinking, show lower levels of alcohol use, are more likely to engage with peers who do not drink and have a higher level of self-efficacy for alcohol refusal [15].

The effect of parental influence on substance use may be equivalent to that of peer influence [6, 37]. Parental alcoholism has also been linked to less than optimal family management. For instance, less parental discipline is instilled by fathers with alcohol use problems when compared to non-alcoholic fathers [38]. Lower levels of emotional support and parental monitoring have also been reported by older children of alcoholic parents [39].

### Study limitations

The focus on respondents from rehabilitation centres may be both advantageous and disadvantageous. Studies involving information rich cases have been associated with useful manifestations of the concepts being studied thereby revealing useful insights while avoiding mere empirical generalizations [40, 41]. The comparatively more informative categorical data allowing for ordinal regression models were used owing to the fact that respondents already had a history of substance use. Various studies have however reported either “protective but reactive interactions” or “classic buffering” effect of protective factors where the different levels of factors may manifest varying extent of risk among respondents [9]. In the current study, instances of non-significant protective effects where other studies report significant associations may, therefore, be attributed to a greater representation of the highest risk levels among the rehabilitation centre participants which may yield protective but reactive interactions whereas lower-risk samples may produce classic buffering effects. For instance, Wootton et al. [42] reported a protective but reactive interaction in their study on a clinical sample of young children, such that the protective role of effective parenting against conduct problems diminished among children with high personality risk [9]. Further sampling is recommended covering clusters of differing socio-demographics and more balanced gender representation. This will enhance the generalizability of these results to other adolescent populations from other geographic regions with different demographic characteristics.

Children responses concerning parent behaviour may also constitute a limitation. It has been postulated that the perception of a child concerning parental behaviour may be more related to the child’s adjustment than is the actual behaviour of his parents. This aspect has however provoked a large quantity of research on children’s perceptions of parental behaviour [17].

## Conclusions

In conclusion, several family management factors with either risk or protective effect on adolescent substance use were outlined. Some factors had either interactive risk or significant protective effect on substance use or lost significance when analysed jointly together with other factors such as controlled variables. It can be surmised that family based prevention programmes based upon significant risk and protective factors reported here may form a cost effective and practical way of dealing not only with prevention of single behaviours but a range of problems emanating from substance abuse such as harder substances, antisocial behaviours and problematic substance use [6]. Other factors should however also be taken into account such as peers, communities, workplace, government policies and services, and the broader economic and social environment which all affect family well-being in an “ecological” manner [6].
